# Supplementary material for: Do Breastfeeding History and Diet Quality Predict Inhibitory Control at Preschool Age?
Source: Nutrients. 2021 Aug 10;13(8):2752. doi: 10.3390/nu13082752 (PMC8398217; doi:10.3390/nu13082752)
Supplement: Supplementary file 1 [file nutrients-13-02752-s001.zip › nutrients-1299318-supplementary.pdf]

Supplementary materials

Table S1. Correlations between mother and partner reports.

	1. BRIEF-P inh (M)	2. BRIEF-P inh (P)	3. BRIEF-P total (M)	4. BRIEF-P total (P)	5. REEF (M)	6. REEF (P)	7. BRIEF-A (M)	8. BRIEF-A (P)
**1**	-							
**2**	0.415**	-						
**3**	0.800**	0.539**	-					
**4**	0.248	0.857**	0.535**	-				
**5**	-0.426**	-0.279*	-0.461**	-0.259	-			
**6**	-0.182	-0.182	-0.181	-0.147	0.407**	-		
**7**	0.359*	0.366*	0.354**	0.318*	0.033	-0.067	-	
**8**	0.256	0.428**	0.324*	0.518*	-0.178	-0.049	0.385**	-

Correlations are denoted as *r*. BRIEF-P inh: Score of the inhibitory control scale of the BRIEF-P; BRIEF-P total: Total score of the BRIEF-P; (M): Questionnaire filled in by mother; (P): Questionnaire filled in by partner.

* indicates a p-value lower than 0.05, ** indicates a p-value lower than 0.01

Table S2. Correlations between potential confounding variables and independent and outcome variables.

	Maternal educational level	BRIEF-A-comp	Child Gender
**Breastfeeding data**			
Exclusive breastfeeding	0.191	0.126	-0.026
Breastfeeding cessation age	0.290*	-0.042	0.098
**Behavioral tasks**			
Flanker	0.106	-0.006	-0.289*
Whisper	-0.002	0.102	-0.124
Gift Wrap	0.063	-0.179	0.396**
Gift Delay	0.433***	-0.270*	0.140
Inhibitory control composite	0.217	-0.250*	0.132
**Questionnaires**			
BRIEF-P-inh	0.008	0.351**	0.157
BRIEF-P	-0.084	0.437 ***	-0.012
REEF	0.080	-0.019	0.021

Correlations are denoted as *r*. BRIEF-P-inh: Score of the inhibitory control scale of the BRIEF-P. BRIEF-A-comp: Composite score of the BRIEF-A filled in by mother and partner. Child gender: 1= boy, 2= girl.

*$p < 0.05$. **$p < 0.01$. ***$p < 0.001$

Table S3. Parameter Estimates and bootstrapped Confidence Intervals for models 7 and 8.

	**B**	**SE**	**Lower CI**	**Upper CI**
**Regression Paths**	Model 7: Exclusive breastfeeding duration → diet quality score → reported executive functioning (BRIEF-P)			
Reported Executive functioning (BRIEF-P)				
Exclusive breastfeeding duration	-0.242	1.198	-2.590	2.107
Diet quality score	1.007	1.484	-1.902	3.917
Parental executive functioning	0.763***	0.056	0.653	0.873
Diet quality score				
Exclusive breastfeeding duration	0.173*	0.070	0.035	0.310
**Mediation effect**	0.174	0.260	-0.336	0.684
**Total effect**	0.695	1.083	-1.429	2.818
**Regression Paths**	Model 8: Breastfeeding cessation age → diet quality score → reported executive functioning (BRIEF-P)			
Reported Executive functioning (BRIEF-P)				
Breastfeeding cessation age	0.338	0.204	-0.061	0.738
Diet quality score	1.143	1.364	-1.531	3.817
Parental executive functioning	0.801***	0.081	0.642	0.960
Maternal educational level	-1.272	1.257	-3.736	1.191
Diet quality score				
Breastfeeding cessation age	0.154	0.131	-0.102	0.411
**Mediation effect**	0.177	0.223	-0.260	0.613
**Total effect**	0.044	1.123	-2.158	2.245

MLR estimator used to calculate parameter estimates, bootstrapping used to calculate bias-corrected confidence intervals. Model 7: X^2 (3)=1.043, p=0.307; CFI = 0.998, RMSEA = 0.024, SRMR = 0.032, n=67. Model 8: X^2 (4)= 1.817, p=0.611, RMSEA = 0.000 , SRMR = 0.046, n=67.

*p < 0.05. ***p < 0.001.

Table S4. Parameter Estimates and bootstrapped Confidence Intervals for Exploratory Models 1, 2, 5, 6, 7, and 8.[†]

	**B**	**SE**	**Lower CI**	**Upper CI**
	Exploratory model 1: Exclusive breastfeeding duration → Diet quality score → Flanker			
**Regression Paths**				
Flanker task				
Exclusive breastfeeding duration	0.059	0.048	-0.035	0.153
Diet quality score	-0.065	0.098	-0.257	0.128
Gender	-0.313	0.211	-0.726	0.099
Diet quality score				
Exclusive breastfeeding duration	0.223**	0.072	0.081	0.365
**Mediation effect**	-0.014	0.021	-0.056	0.028
**Total effect**	-0.269	0.225	-0.710	0.173
	Exploratory model 2: Breastfeeding cessation age → diet quality score → Flanker			
**Regression Paths**				
Flanker task				
Breastfeeding cessation age	0.026*	0.012	0.003	0.048
Diet quality score	-0.062	0.087	-0.234	0.109
Gender	-0.406*	0.188	-0.775	-0.036
Maternal educational level	0.046	0.054	-0.059	0.151
Diet quality score				
Breastfeeding cessation age	0.029	0.018	-0.007	0.065
**Mediation effect**	-0.002	0.003	-0.007	0.003
**Total effect**	-0.336	0.190	-0.709	0.036
	Exploratory model 5: Exclusive breastfeeding duration → Diet quality score → Gift Wrap task			
**Regression Paths**				
Gift Wrap				
Exclusive breastfeeding duration	-0.093	0.048	-0.188	0.001
Diet quality score	0.168	0.092	-0.013	0.349
Gender	0.683**	0.204	0.283	1.082
Diet quality score				
Exclusive breastfeeding duration	0.155*	0.071	0.016	0.293
**Mediation effect**	0.026	0.019	-0.011	0.063
**Total effect**	-0.067	0.044	-0.153	0.018
	Exploratory model 6: Breastfeeding cessation age → Diet quality score → Gift Wrap task			
**Regression Paths**				

Gift Wrap task				
Breastfeeding cessation age	-0.008	0.011	-0.030	0.015
Diet quality score	0.114	0.092	-0.066	0.295
Gender	0.727***	0.196	0.343	1.112
Maternal educational level	0.091	0.059	-0.026	0.207
Diet quality score				
Breastfeeding cessation age	0.156	0.121	-0.082	0.393
**Mediation effect**	0.018	0.019	-0.020	0.056
**Total effect**	0.828***	0.174	0.487	1.169
**Regression Paths**	Exploratory model 7: Exclusive breastfeeding duration → Diet quality score→ Gift Delay task			
Gift Delay task				
Exclusive breastfeeding duration	1.260	1.414	-1.511	4.032
Diet quality score	-1.790	2.187	-6.076	2.497
Parental executive functioning	-0.441*	0.176	-0.788	-0.096
Maternal educational level	6.195**	2.325	1.639	10.752
Diet quality score				
Exclusive breastfeeding duration	0.160*	0.068	0.027	0.294
**Mediation**	-0.287	0.399	-1.069	0.495
**Total model**	6.726**	2.496	1.834	11.619
**Regression Paths**	Exploratory model 8: Breastfeeding cessation age → Diet quality score→ Gift Delay task			
Gift Delay task				
Breastfeeding cessation age	0.603	0.333	-0.049	1.256
Diet quality score	-1.781	2.118	-5.933	2.371
Parental executive functioning	-0.423*	0.167	-0.751	-0.096
Maternal educational level	5.826*	2.255	1.406	10.247
Diet quality score				
Breastfeeding cessation age	0.153	0.125	-0.093	0.398
**Mediation**	-0.272	0.392	-1.040	0.497
**Total model**	0.332	0.443	-0.537	1.200

MLR estimator used to calculate parameter estimates, bootstrapping used to calculate bias-corrected confidence intervals. Exploratory model 1: X^2 (3)= 0.696, p= 0.404, CFI = 1.000, RMSEA = 0.000, SRMR = 0.035, n=52. Exploratory model 2: X^2 (4)= 1.592, p= 0.661; CFI = 1.000, RMSEA = 0.000, SRMR = 0.047, n=52. Exploratory model 5: X^2 (2)= 0.980, p=0.613; CFI = 1.000, RMSEA = 0.000, SRMR = 0.034, n=65. Exploratory model 6: X^2 (3)= 2.079, p=0.721; CFI =

1.000, RMSEA = 0.000, SRMR = 0.047, n=65. Exploratory model 7: X^2 (4)= 1.881, *p*=0.598; CFI = 1.000, RMSEA = 0.000, SRMR = 0.045, n=66. Exploratory model 8: X^2 (4)= 1.765, p=0.623, CFI = 1.000, RMSEA = 0.000, SRMR = 0.044, n=66.

**p* < 0.05. ***p* < 0.01. ****p* < 0.001

† Model 3 (Exclusive breastfeeding duration → Diet quality score → Whisper) and Model 4 (Breastfeeding cessation age → Diet quality score → Whisper) could not be fitted; therefore no parameter estimates could be produced.

Table S5. Parameter Estimates and bootstrapped Confidence Intervals for Exploratory Models 9a, 10a, 11a, 12a, and 14a.†

	B	SE	Lower CI	Upper CI
	Exploratory model 9a: Exclusive breastfeeding duration → Vegetable intake → Executive functioning (BRIEF-P)			
**Regression Paths**				
Executive functioning (BRIEF-P)				
Exclusive breastfeeding duration	0.647	0.872	-1.062	2.356
Vegetable intake	-0.391	0.239	-0.860	0.077
Parental executive functioning	0.363***	0.088	0.190	0.535
Vegetable intake				
Exclusive breastfeeding duration	0.092	0.461	-0.812	0.996
**Mediation effect**	-0.036	0.185	-0.400	0.327
**Total effect**	0.974	0.892	-0.775	2.723
	Exploratory model 10a: Breastfeeding cessation age → Vegetable intake → Executive functioning (BRIEF-P)			
**Regression Paths**				
Executive functioning (BRIEF-P)				
Breastfeeding cessation age	0.341	0.197	-0.044	0.726
Vegetable intake	-0.367	0.232	-0.822	0.089
Parental executive functioning	0.378***	0.088	0.205	0.550
Maternal educational level	-1.004	1.141	-3.247	1.239
Vegetable intake				
Breastfeeding cessation age	-0.033	0.108	-0.246	0.179
**Mediation effect**	0.012	0.040	-0.066	0.090
**Total effect**	-0.274	1.129	-2.486	1.939
	Exploratory model 11a: Exclusive breastfeeding duration → Fruit intake → Executive functioning (BRIEF-P)			
**Regression Paths**				
Executive functioning (BRIEF-P)				
Exclusive breastfeeding duration	0.452	0.932	-1.374	2.278
Fruit intake	0.150	0.222	-0.285	0.584
Parental executive functioning	0.352***	0.087	0.182	0.522
Fruit intake				

Exclusive breastfeeding duration	1.215*	0.538	0.160	2.270
**Mediation effect**	0.178	0.291	-0.392	0.748
**Total effect**	0.981	0.889	-0.761	2.724
**Regression Paths**	Exploratory model 12a: Breastfeeding cessation age → Fruit intake → Executive functioning (BRIEF-P)			
Executive functioning (BRIEF-P)				
Breastfeeding cessation age	0.338	0.201	-0.055	0.731
Fruit intake	0.132	0.202	-0.264	0.528
Parental executive functioning	0.362***	0.090	0.185	0.538
Maternal educational level	-0.940	1.284	-3.457	1.576
Fruit intake				
Breastfeeding cessation age	0.107	0.110	-0.108	0.322
**Mediation**	0.014	0.029	-0.043	0.071
**Total model**	-0.227	1.271	-2.718	2.265
**Regression Paths**	Exploratory model 14a: Breastfeeding cessation age → Snack and Candy intake → Executive functioning (BRIEF-P)			
Executive functioning (BRIEF-P)				
Breastfeeding cessation age	0.347	0.199	-0.043	0.736
Snack and candy intake	-0.076	0.546	-1.146	0.994
Parental executive functioning	0.358***	0.096	0.170	0.545
Maternal educational level	-1.048	1.274	-3.546	1.449
Snack and candy intake				
Breastfeeding cessation age	-0.075	0.043	-0.159	0.009
**Mediation**	0.006	0.042	-0.076	0.087
**Total model**	-0.338	1.260	-2.808	2.132

MLR estimator used to calculate parameter estimates, bootstrapping used to calculate bias-corrected confidence intervals. Exploratory model 9a: $X^2(3)=0.932$, p= 0.334; CFI = 1.000, RMSEA = 0.000, SRMR = 0.033. Results based on N=67. Exploratory model 10a: $X^2(3)= 1.292$, p = 0.863; CFI = 1.000, RMSEA = 0.000, SRMR = 0.038. Results based on N=67. Exploratory model 11a: X^2 (3)=0.684 , *p*= 0.408; CFI = 1.000, RMSEA = 0.000, SRMR = 0.025. Results based on *N*=67. Exploratory model 12a: X² (3)=2.060, p=0.725; CFI = 1.000, RMSEA = 0.000, SRMR = 0.049. Results based on

N=67. Results based on N=66. Exploratory model 14a: X^2 (3)=1.939, p=0.747; CFI = 1.000, RMSEA = 0.000, SRMR = 0.049. Results based on N=67.

*$p < 0.05$. ***$p < 0.001$.

†Model 13a (Exclusive breastfeeding duration → Snack and Candy intake → Executive functioning (BRIEF-P)) could not be fitted; therefore no parameter estimates could be produced.

Table S6. Parameter Estimates and bootstrapped Confidence Intervals for Exploratory Models 9b, 10b, 11b, 13b, and 14b.[†]

	B	SE	Lower CI	Upper CI
**Regression Paths**	Exploratory model 9b: Exclusive breastfeeding duration → Vegetable intake → Executive functioning (REEF)			
Executive functioning (REEF)				
Exclusive breastfeeding duration	-1.959	1.530	-4.958	1.041
Vegetable intake	0.379	0.586	-0.769	1.528
Maternal educational level	3.239	2.258	-1.186	7.665
Vegetable intake				
Exclusive breastfeeding duration	0.077	0.461	-0.826	0.981
**Mediation effect**	0.029	0.173	-0.310	0.368
**Total effect**	1.310	2.446	-3.484	6.103
**Regression Paths**	Exploratory model 10b: Breastfeeding cessation age → Vegetable intake → Executive functioning (REEF)			
Executive functioning (REEF)				
Breastfeeding cessation age	-0.202	0.341	-0.870	0.466
Vegetable intake	0.369	0.596	-0.799	1.538
Maternal educational level	3.087	2.215	-1.255	7.429
Vegetable intake				
Breastfeeding cessation age	-0.006	0.108	-0.218	0.206
**Mediation effect**	-0.014	0.047	-0.107	0.079
**Total effect**	2.870	2.157	-1.358	7.099
**Regression Paths**	Exploratory model 11b: Exclusive breastfeeding duration → Fruit intake → Executive functioning (REEF)			
Executive functioning (REEF)				
Exclusive breastfeeding duration	-2.547	1.713	-5.905	0.812
Fruit intake	0.443	0.381	-0.304	1.191
Maternal educational level	3.732	2.257	-0.692	8.156
Fruit intake				
Exclusive breastfeeding duration	1.263**	0.470	0.341	2.185
**Mediation effect**	0.560	0.588	-0.592	1.712
**Total effect**	1.746	2.383	-2.925	6.417
**Regression Paths**	Exploratory model 13b: Exclusive breastfeeding			

	duration → Snacks and candy intake → Executive functioning (REEF)			
Executive functioning (REEF)				
Exclusive breastfeeding duration	-1.943	1.599	-5.077	1.192
Snacks and candy intake	0.163	0.805	-1.415	1.740
Maternal educational level	3.296	2.199	-1.015	7.606
Fruit intake				
Exclusive breastfeeding duration	-0.310	0.209	-0.720	0.100
**Mediation**	-0.050	0.251	-0.543	0.442
**Total model**	1.303	2.367	-3.336	5.941
**Regression Paths**	Exploratory model 14b: Breastfeeding cessation age → Snack and candy intake → Executive functioning (REEF)			
Executive functioning (REEF)				
Breastfeeding cessation age	-0.230	0.367	-0.949	0.488
Snack and candy intake	0.274	0.784	-1.263	1.811
Maternal educational level	3.176	2.169	-1.075	7.426
Snack and candy intake				
Breastfeeding cessation age	-0.076	0.049	-0.172	0.020
**Mediation**	-0.021	0.062	-0.142	0.100
**Total model**	2.924	2.097	-1.186	7.035

MLR estimator used to calculate parameter estimates, bootstrapping used to calculate bias-corrected confidence intervals. Exploratory model 15: X^2 (2)= 0.859, p = 0.651; CFI = 1.000, RMSEA = 0.000, SRMR = 0.034. Results based on N=67. Exploratory model 16: X^2(3)= 0.204, p = 0.651; CFI = 1.000, RMSEA = 0.000, SRMR = 0.016. Results based on N=67. Exploratory model 17: X^2 (3)=0.791 , p= 0.374; CFI = 1.000, RMSEA = 0.000, SRMR = 0.034. Results based on N=67. Exploratory model 19: X^2 (2)= 0.720, p=0.698; CFI = 1.000, RMSEA = 0.000, SRMR = 0.032. Results based on N=67. Exploratory model 20: X^2 (3)=0.019, p=0.889; CFI = 1.000, RMSEA = 0.000, SRMR = 0.005. Results based on N=67. **p < 0.01.

[†]Model 12b (Breastfeeding cessation age → Fruit intake → Executive functioning (REEF)) could not be fitted; therefore no parameter estimates were produced.
